# Feeding and swallowing function and dysphagia risk in children with posterior fossa tumors: a preliminary Italian cohort study

**DOI:** 10.3389/fonc.2026.1910015

**Published:** 2026-09-01

**Authors:** Marco Tofani, Lavinia Savarese, Bianca Andreozzi, Antonella Cerchiari, Andrea Carai, Carolina Giordani, Giorgia Biondo, Gessica Della Bella, Francesca Pizza, Angela Mastronuzzi

**Affiliations:** 1 Neurorehabilitation and Adapted Physical Activity Day Hospital Unit, Research Area on Innovative Models in Neurorehabilitation, Bambino Gesù Children’s Hospital, IRCCS, Rome, Italy; 2 Department of Life Sciences, Health and Healthcare Professions, Link Campus University, Rome, Italy; 3 Hematology-Oncology, Cell Therapy, Gene Therapies and Hemopoietic Transplant Area Unit, Bambino Gesù Children’s Hospital, IRCCS, Rome, Italy; 4 Pediatric Neurology Unit, Catholic University of Sacred Heart, Rome, Italy; 5 Neurosurgery Unit, Bambino Gesù Children’s Hospital, IRCCS, Rome, Italy; 6 Department of Life Sciences and Public Health, Catholic University of Sacred Heart, Rome, Italy

**Keywords:** chewing abilities, children, dysphagia, feeding, posterior fossa tumors, swallowing

## Abstract

**Introduction:**

Dysphagia is a frequent postoperative complication in children undergoing surgery for posterior fossa tumors (PFTs), yet early swallowing trajectories remain insufficiently characterized despite the growing focus on functional outcomes in pediatric neuro-oncology.

**Methods:**

In this prospective cohort study conducted at a tertiary referral center, feeding and swallowing function were assessed at three standardized time points: preoperatively, one week postoperatively, and four weeks postoperatively using the Pediatric Screening Priority Evaluation for Dysphagia (PS-PED), the American Speech-Language Hearing Association National Outcomes Measurement System (ASHA NOMS) Swallowing Scale, and the Karaduman Chewing Performance Scale (KCPS). Group comparisons were performed according to tumor histology (medulloblastoma [MB], pilocytic astrocytoma [PA], ependymoma [EP]) and extent of resection (partial vs. gross total) using non-parametric methods.

**Results:**

Forty-five children were enrolled. Across all measures, swallowing function followed a dynamic postoperative trajectory, with substantial deterioration at one week and partial recovery at four weeks; however, many children did not return to their baseline status within this timeframe. Tumor characteristics and surgical factors significantly shaped these trajectories: EPs and MBs were associated with higher dysphagia risk and poorer functional oral intake compared with PAs, and partial resections were consistently linked to worse swallowing outcomes. Chewing performance exhibited a similar pattern of acute decline, followed by gradual improvement, with limited differentiation across subgroups.

**Discussion:**

These findings underscore the heterogeneity of early postoperative recovery in PFTs, support the integration of serial multidimensional assessments in routine care, and highlight the need for early, individualized rehabilitation strategies to optimize short-term functional outcomes and inform future prognostic research.

## 1 Introduction

Dysphagia, defined as an impairment in the swallowing process involving oral, pharyngeal, or esophageal phases, is a critical complication in pediatric neuro-oncology, particularly in children with posterior fossa tumors (PFTs). The posterior fossa, located beneath the tentorium cerebelli and above the foramen magnum, contains critical structures, including the brainstem, cerebellum, and cranial nerves IX, X, and XII, which are essential for swallowing function (1, 2). Lesions in this area, whether caused by the tumor itself or by surgical intervention, frequently result in neurological and oropharyngeal dysfunctions that predispose children to dysphagia.

Pediatric brain tumors are the most common solid tumors in childhood, with an overall incidence of 5.7 per 100,000 children (3). PFTs represent 35-45% of these cases and occur at an incidence of 2–3.5 per 100,000 children (4). The most common histologies include medulloblastomas (MB), pilocytic astrocytomas (PA), and ependymomas (EP). Although rare, atypical teratoid/rhabdoid tumors (ATRTs) are also relevant due to their aggressive course (5). Despite advances in surgical and oncological care, the anatomical location of these tumors makes treatment complex and often associated with functional sequelae.

The prevalence of dysphagia following PFT resection varies widely depending on the population studied, the timing of assessment, and the diagnostic methods used. Early reports documented swallowing impairments in 33–73% of children after posterior fossa surgery (6, 7). More recent large-scale evidence, including a systematic review and meta-analysis of 22 studies and 20,921 participants, estimated a pooled prevalence of 21.7% after PFT surgery (4). In specific institutional series, the prevalence is even higher: Wright et al. (5) reported that nearly half of children undergoing posterior fossa resection developed dysphagia requiring referral to speech and language therapy, and Goethe et al. (8) found a rate of 21.8% among almost 200 children treated surgically; risk factors consistently identified include younger age, aggressive histologies, and brainstem compression or invasion.

Dysphagia in this population presents with diverse clinical manifestations. Swallowing impairment may cause aspiration, which is silent in up to 50% of cases (5), and can result in recurrent respiratory infections, aspiration pneumonia, or death (9). Nutritional deficits are also common, frequently necessitating enteral feeding through nasogastric or percutaneous gastrostomy tubes (8). Long-term dependency is not negligible: in Goethe’s cohort, 5.1% of patients remained gastrostomy-dependent one year after surgery. Moreover, dysphagia contributes to prolonged hospitalization and increased healthcare costs (4) while also impairing psychosocial well-being, as eating and drinking are fundamental to childhood autonomy and social participation.

Special consideration is warranted for infants under one year of age. PFTs are rare in this age group, accounting for approximately 17.9–32.4% of intracranial tumors, depending on the series (10, 11). Nevertheless, their impact is disproportionately severe. Picariello et al. (12) described a two-center cohort of 33 infants with PFTs, highlighting that high-grade pathology was the most significant predictor of poor outcome, with a 5-year overall survival of only 55%. Notably, dysphagia was among the presenting features in some cases, alongside classic signs of intracranial hypertension such as vomiting, bulging fontanel, and macrocephaly (13, 14). Surgical management in this very young population is further complicated by physiological fragility, high risk of intraoperative complications, and vulnerability to adjuvant therapy toxicity (9, 15, 16). This makes swallowing rehabilitation even more challenging in infants, as feeding difficulties often overlap with global developmental delays and complex care needs.

Swallowing difficulties can occur in isolation or as part of Posterior Fossa Syndrome, which is characterized by mutism, emotional lability, ataxia, and dysarthria (6, 17). In this syndrome, dysphagia often reflects impairments in motor coordination, cranial nerve function, and oromotor control. Importantly, while the majority of children recover swallowing function within months, recovery is incomplete in a substantial minority: Duan et al. (4) (2024). highlighted that a significant proportion of patients required modified diets beyond three months, and Goethe et al. (8) reported that 27% of affected children had persistent deficits one year after surgery.

From a rehabilitation perspective, several challenges emerge. First, the heterogeneity of tumor types and surgical approaches leads to variable patterns of swallowing dysfunction. Recent studies (5, 18), report increased rates of silent aspiration in children with tumors located in the fourth ventricle and cerebellopontine angle, while EPs and MBs associated with the highest risk of persistent swallowing impairment. Second, assessment pediatric swallowing is challenging: although bedside evaluations are widely used, they may not reliably detect silent aspiration. Videofluoroscopic swallowing studies (VFSS) and fiberoptic endoscopic evaluations of swallowing (FEES) provide more detailed information, but are resource-intensive and not universally available (19). Third, rehabilitation strategies must balance immediate safety, ensuring adequate nutrition and prevention of aspiration, with the progressive reintroduction of oral feeding to promote recovery.

The presence of additional neurological and oncological comorbidities further complicates management. Children with PFTs often experience hydrocephalus, long tract signs, or cranial nerve deficits, each of which may delay swallowing recovery (20–23). Moreover, the burden of oncological treatments, including chemotherapy and radiotherapy, can exacerbate fatigue, reduce neuromuscular reserve, and prolong rehabilitation, emphasizing the need for individualized, multidisciplinary care pathways that integrate neurosurgery, oncology, speech and language therapy, occupational therapy, physiotherapy, and nutritional support (24).

In light of the improved survival rates for pediatric PFTs, functional outcomes such as swallowing safety and efficiency have become central to long-term quality of life. Dysphagia is not only a medical complication but also a determinant of social integration, educational participation, and family burden. The development of a standardized screening tool together with a comprehensive analysis of chewing performance and food consistency can help to deliver appropriate care. Although postoperative dysphagia following PFT resection has been increasingly recognized, available studies have primarily focused on its prevalence, associated risk factors, or longer-term outcomes. Less is known about the early evolution of different components of feeding and swallowing function from the preoperative period through the first weeks after surgery. In particular, prospective studies combining serial assessment of dysphagia risk, functional oral intake, and chewing performance remain limited. Therefore, the present investigation aims to characterize early feeding and swallowing trajectories in children undergoing surgery for posterior fossa tumors by conducting serial assessments before surgery and at two postoperative timepoints. Specifically, the study seeks to (1) quantify peri-operative changes in dysphagia risk, functional oral intake, and chewing performance using validated clinical instruments; (2) compare these trajectories across the most common tumor type, namely MB, PA, and EP; and (3) examine the influence of surgical extent, contrasting partial (PR) versus gross total resection (GTR). By integrating three complementary dimensions of feeding and swallowing function across repeated perioperative assessments, this study provides preliminary evidence on early postoperative trajectories that may support the identification of children at higher risk of persistent impairment and inform individualized rehabilitation pathways.

## 2 Materials and methods

### 2.1 *Study* design *and* setting

This investigation was designed as an observational cohort study following STROBE recommendations. Data were collected prospectively at the IRCCS Bambino Gesù Children’s Hospital, a tertiary pediatric referral center with specialized neuro-oncology and neurorehabilitation services. Children were assessed at three predefined timepoints: baseline (pre-surgery), one week post-surgery, and four weeks post-surgery. These assessments evaluated perioperative changes in feeding and swallowing function.

### 2.2 Participants

All consecutive children diagnosed with a PFT and scheduled for neurosurgical resection between June 2022 and December 2024 were screened for eligibility. Inclusion criteria were: confirmed diagnosis of MB, PA, or EP based on neurosurgical and histopathological evaluation who underwent surgical resection, both PR or GTR; age <18 years; ability to undergo standardized swallowing assessments at all three study timepoints; and no prior brain tumor surgery. Exclusion criteria included: pre-existing neurological or genetic disorders known to affect swallowing (e.g., neuromuscular disease, congenital cranial nerve palsy); medical instability precluding clinical assessment; previous cranial irradiation or chemotherapy for another condition; incomplete follow-up at any assessment point. Furthermore, children diagnosed with ATRTs were excluded because of their low incidence, marked biological heterogeneity, and distinct oncological trajectory, which could have introduced substantial clinical variability within this preliminary cohort. Children with primary brainstem tumors were also excluded, as these lesions are typically not managed through the same surgical approaches used for posterior fossa tumor resection and are often not amenable to surgical removal because of their critical neuroanatomical location and infiltrative growth patterns. Children with incomplete follow-up were excluded before analysis; therefore, no missing outcome data were present in the final dataset.

Eligibility was determined by clinical investigators, and all participants were enrolled consecutively to minimize selection bias. Because posterior fossa tumors are uncommon in the pediatric population, no *a priori* sample size calculation was performed. The study therefore adopted a consecutive sampling strategy, including all eligible patients who met the inclusion criteria during the study period. Demographic and clinical characteristics-including age, sex, tumor histology, tumor location, and extent of resection-were extracted from electronic medical records.

### 2.3 Variables and outcome measures

Three validated clinical instruments were used as primary outcome measures, each selected for its established reliability, clinical relevance, and suitability for monitoring perioperative changes in pediatric feeding and swallowing.

#### 2.3.1 Pediatric Screening Priority Evaluation for Dysphagia

The PS-PED (25) is a 14-item dichotomous screening tool designed to identify children at risk of dysphagia across multiple domains, including neurological status, structural abnormalities, respiratory comorbidities, nutritional indicators, and feeding-related behaviors. Each item is scored as present or absent, yielding a total score ranging from 0 to 14; values ≥6 have been shown to indicate elevated dysphagia risk (26). The instrument demonstrates strong measurement properties, with reported inter-rater reliability coefficients above 0.80, supporting consistency across clinicians, and good construct validity, as PS-PED scores correlate with instrumental swallowing assessments and clinical severity markers. Its multidimensional structure enhances sensitivity to early clinical deterioration, making it well-suited to detect acute postoperative changes in children with neurological or neurosurgical conditions. In the present study, the PS-PED was administered at baseline, one week, and four weeks after surgery to quantify global dysphagia risk across the perioperative timeline.

#### 2.3.2 ASHA NOMS swallowing level

The American Speech-Language-Hearing Association (ASHA)’s National Outcomes Measurement System (NOMS) swallowing scale (27) is a 7-level ordinal measure developed to capture functional oral intake and swallowing safety in clinical practice. Levels range from 1 (severe impairment requiring non-oral feeding) to 7 (independent, age-appropriate swallowing), providing a structured classification of consistency tolerance, need for supervision, compensatory strategies, and aspiration risk. The scale has demonstrated excellent content and face validity, having been developed through national expert consensus, and good responsiveness to functional change, making it frequently used in pediatric rehabilitation and dysphagia research. Studies report moderate-to-high inter-rater agreement despite its clinically oriented structure, and it is recommended as an outcome indicator in functional rehabilitation programs. In this study, ASHA NOMS was used to evaluate the child’s ability to safely manage food and liquid consistencies at the three study timepoints.

#### 2.3.3 Karaduman Chewing Performance Scale

The KCPS (28) is a 5-level ordinal scale (0–4) assessing chewing performance, with higher values indicating more severe impairment. The scale evaluates coordination, bolus formation, lip closure, rotary jaw movements, and the overall efficiency of mastication. It was specifically developed for pediatric populations and has shown excellent inter-rater reliability, with agreement levels exceeding 0.85 across trained raters. For the present study, the Italian version of the KCPS was used (29).

### 2.4 Data analysis

All outcome measures were administered by experienced speech and language pathologists with a specialization in pediatric dysphagia, following standardized protocols to ensure scoring consistency across examiners. Baseline evaluations were conducted within 24 hours before surgery, while postoperative assessments at one and four weeks were integrated into routine clinical follow-up or inpatient rehabilitation pathways.

Tumor type-classified as MB, PA, or EP, and the extent of surgical resection (GTR vs PR) were treated as exposure variables. These clinical factors were selected based on their known influence on PFTs postoperative neurological outcomes, and were therefore hypothesized to modulate swallowing and feeding trajectories.

To reduce selection bias, all eligible patients were enrolled consecutively during the study period. The use of validated instruments minimized measurement error, and although blinding of assessors was not feasible in this clinical context, uniform administration procedures were applied to limit observer-related variability. Missing data were minimized by embedding assessments within standard care schedules, and cases lacking complete follow-up were excluded according to predefined criteria.

The statistical analysis aimed to determine whether significant differences existed in swallowing and feeding outcomes across tumor type (MB, PA, EP) and extent of surgical resection (PR vs GTR). Primary variables included the total PS-PED score, ASHA NOMS swallowing level, and KCPS score, each examined at baseline, one week, and four weeks to characterize short-term postoperative trajectories. Analyses began with descriptive statistics. To provide a comprehensive representation of the data, both parametric and distribution-free indices were reported. Means and standard deviations (SD) were used to summarize central tendency and variability under the assumption of approximate normality, while medians and interquartile ranges (IQR) offered robust estimates for skewed or heterogeneous distributions. This dual reporting was particularly relevant given the small group sizes and clinical variability inherent to pediatric neuro-oncology populations. The distribution of each variable was assessed using the Shapiro–Wilk test (30). Because several measures deviated significantly from normality (p < 0.05), non-parametric statistical tests were selected for inferential analyses. Group differences across the three tumor types were examined using the Kruskal–Wallis test, which evaluates differences in rank distributions without assuming normality or homoscedasticity. When the Kruskal–Wallis test (31) indicated a significant effect, *post hoc* pairwise comparisons were performed using the Mann–Whitney U test (32). For comparisons involving two groups (PR vs GTR), overall group differences were assessed using the Kruskal–Wallis test and confirmed by Mann–Whitney U pairwise comparisons.

To limit Type I error inflation due to multiple comparisons, the Bonferroni correction was employed (33, 34). When three *post hoc* tests were conducted, the significance threshold was adjusted to α = 0.017 (0.05/3). Although conservative and associated with reduced statistical power, this approach ensured that detected group differences were unlikely to result from chance variation. Effect sizes were reported as Kendall’s coefficient of concordance (W) for Friedman tests and epsilon-squared (ϵ²) for Kruskal–Wallis analyses. All tests were two-tailed. A p-value < 0.05 was considered significant for single comparisons, unless otherwise adjusted by the Bonferroni criteria.

## 3 Results

The sample consists of a total of 45 children (68.9% male) with a mean (SD) age of 7.57 (4.7). Main characteristics are summarized in **Table 1**.

**Table 1:** Sample characteristics.

Characteristics	Values
Age mean (SD)	7.57 (4.7)
Sex	N (%)
Female	14 (31.1)
Male	41 (68.9)
School	N (%)
No schooling due to the age	6 (13.3)
Kindergarten	15 (33.3)
Primary school	16 (35.6)
Secondary school	8 (17.8)
Tumor type	N (%)
MB	17 (37.8)
PA	15 (33.3)
EP	13 (28.9)
Resection	N (%)
GTR	15 (33.3)
PR	30 (66.7)

Concerning the screening for dysphagia using PS-PED, no children were found at-risk at baseline. Post-surgery, 47% of children were at risk, and four weeks post-surgery, the proportion decreased to 26.7%. Detailed information for each item of the PS-PED for each time interval is reported in **Table 2**.

**Table 2:** PS-PED scores for each time interval.

PS-PED	BASELINE		1 WEEK		4 WEEKS	
ITEMS	YES	NO	YES	NO	YES	NO
1 Neurological diagnosis	45 (100.0)	0 (0.0)	44 (97.8)	1 (2.2)	45 (100.0)	0 (0.0)
2 Epilepsy medications	0 (0.0)	45 (100.0)	9 (20.0)	36 (80.0)	7 (15.6)	38 (84.4)
3 Heart disease	0 (0.0)	45 (100.0)	0 (0.0)	45 (100.0)	0 (0.0)	45 (100.0)
4 Structural anomalies of the digestive/respiratory systems	2 (4.4)	43 (95.6)	7 (15.6)	38 (84.4)	10 (22.2)	35 (77.8)
5 Tracheal tube	0 (0.0)	45 (100.0)	1 (2.2)	44 (97.8)	1 (2.2)	44 (97.8)
6 Decreased alertness	2 (4.4)	43 (95.6)	12 (26.7)	33 (73.3)	3 (6.7)	42 (93.3)
7 Malnutrition and/or poor growth	7 (15.6)	38 (84.4)	2 (4.4)	43 (95.6)	8 (17.8)	37 (82.2)
8 Recurrent respiratory tract infections	3 (6.7)	42 (93.3)	4 (8.9)	41 (91.1)	4 (8.9)	41 (91.1)
9 Use of suction machine/aspirator	0 (0.0)	45 (100.0)	17 (37.8)	28 (62.2)	10 (22.2)	35 (77.8)
10 Lack of head control and/or postural instability	7 (15.6)	38 (84.4)	25 (55.6)	20 (44.4)	16 (35.6)	29 (64.4)
11 Gastrointestinal diseases (gag reflex, vomit, constipation, GERD)	28 (62.2)	17 (37.8)	2 (4.4)	43 (95.6)	1 (2.2)	44 (97.8)
12 Parenteral/Enteral nutrition (NG tube, gastrostomy, etc.)	0 (0.0)	45 (100.0)	20 (44.4)	25 (55.6)	12 (26.7)	33 (73.3)
13 Feeding with inappropriate food consistency for the developmental stage	1 (2.2)	44 (97.8)	24 (53.3)	21 (46.7)	17 (37.8)	28 (62.2)
14 Prolonged mealtime (> 50 min)	3 (6.7)	42 (93.3)	25 (55.6)	20 (44.4)	17 (37.8)	28 (62.2)
Risk of Dysphagia	n (%)		n (%)		n (%)	
PS-PED Score 5 or less (low risk)	45 (100)		24 (53.3)		33 (73.3)	
PS-PED Score 6 or more (at risk)	0 (0.0)		21 (46.7)		12 (26.7)	

**Table 3** summarizes changes in dysphagia risk, swallowing function, and chewing performance from baseline to one and four weeks after surgery. Significant overall changes over time were observed for all three outcome measures. The magnitude of the overall change was small for the PS-PED and moderate-to-large for both the ASHA NOMS and KCPS.

**Table 3:** Comparison of dysphagia risk, food consistency, and chewing performance.

Outcome	Baseline, mean (SD); median [IQR]	1 week, mean (SD); median [IQR]	4 weeks, mean (SD); median [IQR]	Friedman χ²(2), p	Kendall’s W	Baseline vs 1 week, adjusted p; r	1 vs 4 weeks, adjusted p; r	Baseline vs 4 weeks, adjusted p; r
PS-PED	2.11 (0.86); 2 [2–3]	4.27 (3.10); 4 [1–7]	3.36 (3.03); 2 [1–7]	7.51, 0.023	0.08	<0.001; 0.58	0.005; 0.47	0.105; 0.31
ASHA NOMS	6.93 (0.45); 7 [7–7]	4.47 (2.42); 4 [2–7]	5.40 (2.16); 7 [3–7]	41.52, <0.001	0.46	<0.001; 0.68	0.001; 0.53	<0.001; 0.54
KCPS	0.09 (0.60); 0 [0–0]	1.91 (1.88); 2 [0–4]	1.16 (1.55); 0 [0–3]	42.00, <0.001	0.47	<0.001; 0.67	<0.001; 0.56	<0.001; 0.56

*Post hoc* analyses showed a significant increase in PS-PED scores from baseline to one week, indicating greater dysphagia risk in the early postoperative period, followed by a significant reduction between one and four weeks. The difference between baseline and four weeks was no longer significant after Bonferroni correction. ASHA NOMS scores decreased significantly between baseline and one week and subsequently improved between one and four weeks. However, scores at four weeks remained significantly lower than at baseline. Similarly, KCPS scores increased significantly at one week and decreased between one and four weeks, although chewing performance at four weeks remained significantly poorer than at baseline.

### 3.1 Group differences by tumor type

**Table 4** summarizes between-group comparisons for PS-PED, ASHA NOMS, and KCPS scores at baseline, 1 week, and 4 weeks after surgery. Significant differences between tumor groups were observed for PS-PED scores at 4 weeks (H = 6.70, p = 0.035), for ASHA NOMS scores at 1 week (H = 8.95, p = 0.011) and 4 weeks (H = 8.90, p = 0.012), and for KCPS scores at 1 week (H = 9.16, p = 0.010) and 4 weeks (H = 7.37, p = 0.025). Bonferroni-adjusted pairwise comparisons identified a significant difference between the PA and E groups for ASHA NOMS at 1 week (p = 0.015). No significant pairwise differences remained after Bonferroni correction for PS-PED at 4 weeks or for KCPS at either postoperative assessment.

**Table 4:** Group differences by tumor type: medulloblastoma group (M), pilocytic astrocytoma subgroup (PA), and ependymoma subgroup (E).

Outcome	Time	M (n = 17) mean (SD); median [IQR]	PA (n = 15) mean (SD); median [IQR]	E (n = 13) mean (SD); median [IQR]	Kruskal–Wallis H (p)
PS-PED	Baseline	2.20 (0.70); 2 [2–2]	1.85 (1.14); 2 [1–2]	2.25 (0.75); 2 [2–3]	4.00 (0.135)
	1 week	4.70 (3.03); 5 [1–7]	2.46 (2.82); 1 [1–2]	5.50 (2.84); 6 [5–7]	5.56 (0.062)
	4 weeks	3.15 (2.80); 2 [1–4]	2.00 (2.74); 1 [1–2]	5.17 (3.04); 7 [2–7]	6.70 (0.035)
ASHA NOMS	Baseline	7.00 (0.00); 7 [7–7]	7.00 (0.00); 7 [7–7]	6.75 (0.87); 7 [7–7]	2.75 (0.253)
	1 week	4.10 (2.34); 3 [2–7]	6.15 (2.08); 7 [7–7]	3.25 (2.01); 2 [2–4]	8.95 (0.011)
	4 weeks	5.50 (1.96); 7 [4–7]	6.54 (1.66); 7 [7–7]	4.00 (2.30); 3 [2–7]	8.90 (0.012)
KCPS	Baseline	0.00 (0.00); 0 [0–0]	0.00 (0.00); 0 [0–0]	0.33 (1.15); 0 [0–0]	2.75 (0.253)
	1 week	2.30 (1.87); 3 [0–4]	0.62 (1.33); 0 [0–0]	2.67 (1.83); 4 [1–4]	9.16 (0.010)
	4 weeks	0.95 (1.36); 0 [0–2]	0.46 (1.13); 0 [0–0]	2.25 (1.76); 3 [0–4]	7.37 (0.025)

M, medulloblastoma; PA, pilocytic astrocytoma; E, ependymoma.

### 3.2 Group differences by extent of resection

**Table 5** presents the results of the Kruskal–Wallis analysis and the Mann–Whitney U *post hoc* comparisons for PR versus GTR across PS-PED, ASHA NOMS, and KCPS at baseline, one week, and four weeks. For PS-PED, significant between-group differences were observed at 1 week (H = 8.00, *p* = 0.01, ϵ² = 0.16) and 4 weeks (H = 10.00, *p* = 0.01, ϵ² = 0.20), indicating moderate effect sizes, with children who underwent PR showing higher dysphagia risk than those who underwent GTR. Similarly, ASHA NOMS scores differed significantly between groups at baseline (H = 5.20, *p* = 0.02, ϵ² = 0.098), 1 week (H = 7.20, *p* = 0.01, ϵ² = 0.14), and 4 weeks (H = 8.50, *p* = 0.01, ϵ² = 0.17), corresponding to small-to-moderate effect sizes and reflecting lower swallowing function in the PR group. Pairwise Mann–Whitney U comparisons were consistent with the overall Kruskal–Wallis analyses (all *p* ≤ 0.02). In contrast, no significant between-group differences were observed for KCPS at any assessment, with consistently small effect sizes (all ϵ² ≤ 0.06).

**Table 5:** Group differences among children who underwent partial (n 30) and total (n 15) surgical resection.

Outcome	Timepoint	Partial mean (SD)	Total mean (SD)	Median (PR, GTR)	H (p)	U (p) PR vs GTR
PS-PED	Pre-test	2.32 (0.99)	2.00 (0.86)	2, 2	1.60 (0.20)	310.0 (0.21)
	1 week	5.10 (3.04)	3.60 (3.04)	6, 2	8.00 (0.01*)	240.0 (<0.01*)
	4 weeks	3.80 (3.24)	3.00 (2.87)	2, 1	10.00 (0.01*)	250.0 (<0.01*)
ASHANOMS	Pre-test	6.85 (0.67)	7.00 (0.00)	7, 7	5.20 (0.02*)	195.0 (0.02*)
	1 week	3.85 (2.27)	4.96 (2.46)	2, 7	7.20 (0.01*)	220.0 (<0.01*)
	4 weeks	5.20 (2.32)	5.56 (2.06)	7, 7	8.50 (0.01*)	240.0 (<0.01*)
KCPS	Pre-test	0.20 (0.89)	0.00 (0.00)	0, 0	3.80 (0.05)	230.0 (0.05)
	1 week	2.40 (1.82)	1.52 (1.87)	3.5, 0	3.20 (0.07)	260.0 (0.07)
	4 weeks	1.40 (1.64)	0.96 (1.49)	0.5, 0	2.50 (0.11)	270.0 (0.11)

## 4 Discussion

Understanding swallowing trajectories in PFTs is essential for anticipating postoperative complications and guiding early rehabilitation. By analyzing repeated assessments and stratifying outcomes by tumor type and extent of resection, the present study delineates the specific challenges and recovery patterns observed in this population.

At baseline (T0), all children were classified as being at low risk of dysphagia according to the PS-PED global score. This finding might appear counterintuitive given the well-established pathophysiology of PFTs, which often affect structures directly involved in the coordination of swallowing. Even before surgical intervention, these factors can produce subtle impairments that do not immediately translate into overt dysphagia, but may manifest as reduced nutritional efficiency, delayed gastric emptying, or impaired autonomic control of gastrointestinal function (35). In line with these mechanisms, a proportion of children in our cohort presented with malnutrition or insufficient weight gain (15.6%) and with gastrointestinal disturbances such as recurrent vomiting, constipation, or gastroesophageal reflux (62.2%). These manifestations, although not sufficient to elevate the PS-PED score above the threshold for dysphagia risk, are consistent with the broader neurological and systemic impact of PFTs (36). They may represent early, prodromal expressions of compromised neurophysiological regulation, highlighting that reliance on a global screening score alone may mask the presence of underlying vulnerabilities.

The dramatic change observed at one-week post-treatment (T1) mirrors the acute physiological consequences of surgery in the posterior fossa. As expected, this timepoint was characterized by a sharp increase in the frequency of several PS-PED items, alongside a substantial rise in the number of children classified as “at risk” of dysphagia (46.7%), in line with other studies about the proportion of risk of dysphagia (4, 5, 16, 37). In the immediate post-operative phase, edema of the cerebellar peduncles and the dorsal medulla, transient cranial nerve dysfunction, and reduced consciousness due to medication or anesthetic effects can severely disrupt swallowing coordination. Consistent with these mechanisms, decreased alertness, postural instability, reliance on suction for secretion management, and the need for enteral nutrition became far more prevalent at this stage. The marked increase in prolonged mealtime and use of inappropriate food consistencies further reflects the mechanical and coordinative difficulties commonly observed in this acute window. These findings align with clinical observations reported in the literature, where children undergoing resection of PFTs frequently demonstrate transient but significant deterioration in oral motor control and airway protection in the first post-operative days (38, 39).

By the four-week assessment (T2), the overall profile suggested a partial recovery, and the proportion of children classified at dysphagia risk had fallen to 26.7%. Nevertheless, recovery was not homogeneous, and a subset of children continued to exhibit persistent impairments. Structural anomalies, nutritional problems, postural instability, prolonged mealtime, and the use of inappropriate food consistencies remained evident in a considerable proportion of the cohort. This finding is consistent with the clinical heterogeneity of PFTs, where children with persistent cranial nerve deficits or cerebellar dysfunction often present with prolonged oral motor incoordination, fatigue during feeding, and limited capacity to progress safely toward age-appropriate textures. The variability observed in our cohort reflects the diversity of tumor types, and surgical resection as the distinct recovery trajectories associated with FPTs (38, 39).

Taken together, the results highlight three overarching considerations about feeding and swallowing in children with PFTs. First, a low PS-PED score at baseline does not preclude the presence of clinically relevant early warning signs, which may reflect the broader neurophysiological impact of the tumor before treatment. Second, the acute post-operative phase represents a period of heightened vulnerability, in which structural, neurological, and systemic factors converge to produce a notable rise in dysphagia-related risks. Third, although partial improvement is evident by four weeks, a substantial proportion of children continues to require targeted monitoring, nutritional management, and rehabilitative support. This underscores the importance of serial assessment rather than single-timepoint screening, as well as the integration of clinical assessment with structured tools to inform individualized, timely intervention pathways.

### 4.1 Longitudinal changes across PS-PED, ASHA NOMS, and KCPS

The longitudinal analysis conducted with the Friedman test demonstrates that all three measures (PS-PED, ASHA NOMS, and KCPS) changed significantly across the three assessment points, indicating that feeding and swallowing abilities in children with PFTs evolve dynamically throughout the perioperative period. Although the direction and magnitude of change varied between instruments, the overall pattern is clinically consistent with the expected postoperative trajectory of the target population.

For the PS-PED, the increase observed at one week reflects a clear escalation in dysphagia risk during the acute postoperative phase, followed by a partial decline by four weeks. The Friedman test confirmed a significant overall change (H = 7.51, p = 0.02), and *post-hoc* analyses showed that both the pre–1 week and 1–4 weeks comparisons reached significance after Bonferroni correction, while the pre–4 weeks contrast did not. This indicates that the most pronounced deterioration emerges immediately after surgery, whereas by the four-week follow-up, many children show partial recovery, returning toward, but not fully matching, their preoperative condition. The absence of a significant pre–4–week contrast supports the descriptive data showing that the proportion of children classified as “at risk” decreases substantially from week 1 to week 4. Clinically, this pattern coheres with the expected postoperative swelling, transient cranial nerve dysfunction, and initial reduction in postural stability, which tend to improve with time as inflammation resolves and neurological function begins to re-stabilize.

A similar, yet more clearly defined, temporal pattern is seen for the ASHA NOMS swallowing level, which assesses the functional consistency that a child can safely manage. Here, the Friedman test revealed a highly significant overall effect (H = 41.51, p < 0.001), with *post-hoc* results showing significant differences across all time comparisons after correction. The sharp deterioration from baseline to one week reflects the decline in safe oral intake following posterior fossa surgery, may be characterized by reduced airway protection, limited endurance, postoperative nausea, and disruption of coordinated oro-pharyngeal movements. By four weeks, the median ASHA NOMS score returned to 7, the same level observed at baseline; however, the distribution remained broader, indicating that a subset of children continued to display functional limitations. This suggests that, while many children recover the ability to handle appropriate textures within one month, a proportion remains vulnerable due to persistent deficits in cranial nerve function or cerebellar coordination (40, 41).

The KCPS, which captures chewing performance, exhibited the most marked postoperative deterioration, with a Friedman H of 42.00 (p < 0.001). Chewing function was almost intact at baseline, consistent with the limited presence of overt oral motor symptoms before surgery. However, after one week, the mean KCPS score increased substantially, indicating more pronounced challenges in masticatory coordination, bolus formation, and endurance. *Post hoc* analyses demonstrated significant differences across all pairwise comparisons after Bonferroni correction. Chewing performance deteriorated markedly during the first postoperative week and subsequently improved by the four-week follow-up, although it had not yet returned to preoperative levels.

Taken together, the inferential findings provide a coherent picture of perioperative swallowing dynamics in children with PFTs. The acute postoperative period emerges as the phase of greatest impairment, marked by a convergence of elevated dysphagia risk, reduced tolerance to oral consistencies, and impaired chewing function. By four weeks, improvements are detectable across all measures, though not uniformly, and not all children returned to preoperative levels. Overall, the trajectories observed across PS-PED, ASHA NOMS, and KCPS support the clinical need for serial assessment and individualized rehabilitation, emphasizing that postoperative feeding challenges in PFTs are not only frequent but also follow distinct patterns of deterioration and recovery.

### 4.2 Group differences by tumor type

The comparison of swallowing and feeding outcomes across tumor types showed distinct patterns that correspond closely to the known anatomical and surgical characteristics of MBs, PAs, and EPs. With respect to PS-PED scores, the absence of group differences at baseline (H = 4.00, p = 0.135) suggests that preoperative dysphagia risk was comparable across tumor type. Mean values at this stage were uniformly low: 2.20 for MB, 1.85 for PA, and 2.25 for EP, indicating that despite differences in tumor type, children tended to present with similar functional profiles before surgery. The situation changed in the postoperative period. One week after treatment, children with EP displayed a more pronounced elevation in dysphagia risk (mean PS-PED = 5.50) compared with PA (2.46) and MB (4.70), with the overall group effect approaching significance (H = 5.56, p = 0.06, ϵ² = 0.08). By the four-week follow-up, this divergence had become statistically significant (H = 6.70, p = 0.03, ϵ² = 0.10), with mean PS-PED scores of 5.17 in EP, 3.15 in MB, and 2.00 in PA. However, none of the pairwise comparisons remained statistically significant after Bonferroni correction, suggesting that the overall group effect should be interpreted cautiously and may reflect variability in postoperative recovery across tumor types rather than a clearly defined difference between two specific groups (15, 42–44).

The ASHA NOMS trajectory provides a complementary perspective. No significant differences were observed before surgery (H = 2.75, p = 0.253). Mean scores were 7.00 in both MB and PA and 6.75 in EP, indicating broadly comparable baseline swallowing function. The early postoperative phase showed greater divergence: at one week, median ASHA NOMS scores were 3 for MB, 7 for PA, and 2 for EP, with corresponding mean scores of 4.10, 6.15, and 3.25, respectively. The overall between-group difference was statistically significant (H = 8.95, p = 0.0, ϵ² = 0.16), and Bonferroni-adjusted *post hoc* analysis identified a significant difference between PA and EP (adjusted p = 0.015). At four weeks, significant overall differences remained (H = 8.90, p = 0.012, ϵ² = 0.16), with mean scores of 5.50 for MB, 6.54 for PA, and 4.00 for EP. However, no individual pairwise comparison remained statistically significant after Bonferroni correction at this assessment, indicating greater variability in recovery within and between groups. These findings may reflect differences in tumor location, surgical complexity, and postoperative recovery trajectories across posterior fossa tumor subtypes (45, 46).

Chewing ability followed yet another pattern. At baseline no significant differences were observed across tumor types (H = 2.75, *p* = 0.253, ϵ² = 0.018). At one week, significant overall differences emerged (H = 9.16, *p* = 0.010, ϵ² = 0.16), with mean KCPS scores of 2.30 for MB, 0.62 for PA, and 2.67 for EP. At four weeks, the overall group difference remained significant (H = 7.37, *p* = 0.025, ϵ² = 0.12), with mean KCPS scores of 0.95 for MB, 0.46 for PA, and 2.25 for EP. However, none of the pairwise comparisons remained statistically significant after Bonferroni correction, suggesting that the observed overall differences reflected variability across all three tumor groups rather than a single dominant comparison. This delayed divergence suggests that postoperative chewing recovery may differ across tumor types (47, 48). However, because anatomical tumor location was not specifically analyzed, the mechanisms underlying these differences cannot be determined from the present study.

The *post hoc* analyses further refined the interpretation of the overall group differences. Following Bonferroni correction, only one pairwise comparison remained statistically significant, showing lower ASHA NOMS scores in children with EP than in those with PA at one week after surgery. Although overall group differences were also observed for PS-PED at four weeks and KCPS at one and four weeks, no individual pairwise comparison remained significant after adjustment for multiple testing. Nevertheless, descriptive data consistently indicated a more favorable postoperative functional profile in children with PA, whereas children with EP generally showed greater swallowing and chewing impairment during the early postoperative period. These findings suggest that postoperative swallowing recovery may differ according to tumor type; however, larger studies are needed to confirm these patterns and better characterize differences between individual diagnostic groups.

### 4.3 Group differences by extent of resection

The comparison between PR and GTR resection revealed a consistent pattern across the swallowing-related measures, with more pronounced difficulties observed in children who underwent PR of the lesion. This trend was already evident at baseline, although not yet statistically significant. Before surgery, PS-PED scores were slightly higher in the PR group (mean = 2.32, SD = 0.99) compared with the GTR group (mean = 2.00, SD = 0.86), and ASHA NOMS values showed a similar directional difference, with children scheduled for PR displaying marginally lower functional levels (mean = 6.85 vs 7.00). These baseline contrasts reflect the clinical reality that surgeons frequently opt for PR in cases where lesion location, vascular involvement, or proximity to the brainstem makes complete resection high-risk. In such cases, preoperative function may already be subtly affected by more extensive local invasion or compression, a hypothesis consistent with the small but detectable baseline differences observed.

The postoperative phase sharpened these contrasts considerably. One-week post-surgery, PS-PED scores diverged markedly between the two groups, with the PR group reaching a mean of 5.10 (SD = 3.04), compared with 3.60 (SD = 3.04) in the GTR resection group. The Kruskal–Wallis test confirmed a significant group effect at this point (H = 8.00, p = 0.01), and the Mann–Whitney U comparison reinforced this difference (U = 240.0, p < 0.01). The same pattern emerged for ASHA NOMS, where children with PR demonstrated more compromised levels of functional oral intake (mean = 3.85, SD = 2.27) than those who underwent complete tumor removal (mean = 4.96, SD = 2.46). The underlying mechanisms are clinically plausible. PR is generally undertaken in cases where the tumor involves, or is closely adherent to, critical neuroanatomical structures such as brainstem nuclei, cranial nerve pathways, or cerebellar circuits, rendering complete resection unsafe. In these situations, surgical strategy is guided by the need to minimize the risk of permanent neurological damage. Consequently, postoperative swallowing impairment in this group is more appropriately interpreted as a reflection of the underlying anatomical complexity and the necessity of preserving these eloquent regions, rather than as a direct consequence of residual tumor tissue.

At the four-week evaluation, the group differences persisted. PS-PED scores remained higher in the PR group (mean = 3.80 vs 3.00; H = 10.00, p = 0.01; U = 250.0, p < 0.01), indicating that dysphagia risk had not fully converged between groups despite partial postoperative recovery. ASHA NOMS scores also continued to favor the GTR group (means = 5.20 vs 5.56; H = 8.50, p = 0.01; U = 240.0, p < 0.01), even though both groups showed improvement compared with the acute postoperative phase. These findings suggest that the need for a more conservative surgical approach is associated with different postoperative recovery trajectories. However, this association is likely to reflect the greater anatomical complexity of tumors requiring PR rather than the presence of residual disease per se.

KCPS scores showed a different pattern. At none of the timepoints did chewing performance differ significantly between the groups, although the partial resection group tended to display slightly higher mean values, particularly at one week (2.40 vs 1.52; H = 3.20, p = 0.07 ϵ² = 0.05). This finding may suggest that postoperative chewing performance is influenced less by the extent of surgical resection itself and more by other clinical factors, such as tumor characteristics and individual postoperative recovery patterns.

### 4.4 Limitations

This study has several limitations that should be considered when interpreting the findings. First, the sample size was relatively small, particularly for children presenting clinically significant feeding and swallowing disorders. Given the rarity and heterogeneity of pediatric PFTs, the present cohort should be considered exploratory, and the subgroup analyses interpreted with caution.

Another important limitation is that tumor localization was not analyzed in detail in relation to swallowing outcomes. The study did not specifically distinguish lesions involving structures such as the fourth ventricle, cerebellopontine angle, or areas adjacent to the brainstem. As a result, it was not possible to determine the relative contribution of tumor histology and precise neuroanatomical involvement to postoperative dysphagia. Future studies should integrate both histological and anatomical variables to better understand the mechanisms underlying feeding and swallowing impairments in this population. Other clinical factors that may have influenced postoperative swallowing outcomes, including hydrocephalus, cranial nerve deficits, postoperative complications, and adjuvant treatments, were not specifically accounted for in the analyses. Given the limited sample size, their independent contribution to the observed trajectories could not be explored.

The relationship between dysphagia and posterior fossa syndrome/cerebellar mutism syndrome was also not specifically investigated. Since cerebellar mutism syndrome may significantly influence oral motor and swallowing function during the postoperative period, future research should further explore its association with dysphagia trajectories and neurofunctional recovery.

In addition, instrumental swallowing assessments such as videofluoroscopic swallowing study or fiberoptic endoscopic evaluation of swallowing were not systematically performed. Although the study relied on standardized clinical tools integrated into routine care, instrumental evaluations could provide additional information regarding silent aspiration and pharyngeal phase impairments. Future studies integrating both clinical and instrumental swallowing assessments are warranted to provide a more comprehensive characterization of postoperative dysphagia.

Finally, the four-week follow-up captures only the early postoperative phase and does not allow conclusions regarding longer-term swallowing recovery. Longer follow-up is needed to determine whether the functional changes observed during the first postoperative month persist or resolve over time.

## 5 Conclusions

This study demonstrates that swallowing function in children undergoing surgery for PFTs varies considerably in the early peri-operative period and is affected by both tumor- and treatment-related factors. Across PS-PED, ASHA NOMS, and KCPS, we observed a consistent pattern of acute deterioration at one week followed by partial recovery at four weeks, with many children failing to return to their preoperative status. Group comparisons indicated that tumor type and extent of resection meaningfully influence these trajectories. Clinically, these findings support the routine use of serial, multidimensional assessment rather than single-timepoint screening, and reinforce the need for early, individualized rehabilitation pathways that integrate medical, nutritional, and neurorehabilitation expertise. Future multicenter studies with extended follow-up and integrated clinical–instrumental protocols are warranted to refine prognostic models and inform targeted interventions aimed at improving long-term swallowing outcomes and functional participation in this vulnerable population.

## Data Availability

The original contributions presented in the study are included in the article/supplementary material. Further inquiries can be directed to the corresponding author.
